# Theoretical and Therapeutic Implications of the Spasticity-Plus Syndrome Model in Multiple Sclerosis

**DOI:** 10.3389/fneur.2021.802918

**Published:** 2022-02-07

**Authors:** Antonio Bruno, Ettore Dolcetti, Diego Centonze

**Affiliations:** ^1^Synaptic Immunopathology Lab, Department of Systems Medicine, Tor Vergata University, Rome, Italy; ^2^Department of Neurorehabilitation, Unit of Neurology, Istituto di Ricovero e Cura a Carattere Scientifico (IRCCS) Neuromed, Pozzilli, Italy

**Keywords:** multiple sclerosis, spasticity, cannabinoid, symptoms therapy, axonal transmission

## Abstract

In patients with multiple sclerosis (MS), a typical pattern of muscle tone alteration, known as spasticity, is frequently observed in combination with other signs or symptoms such as spasms, cramps, pain, bladder dysfunction, sleep disturbances, fatigue, and tremor. Recently, the concept of spasticity-plus syndrome (SPS) has been proposed to take into account the frequent coexistence of all these complaints in patients with MS and a common pathophysiological basis for this putative new clinical entity has been proposed. Muscle tone, sleep, bladder function, and the pain pathway are controlled by cannabinoid CB1 (CB1R) and CB2 receptors (CB2R) that are particularly enriched in the brainstem. Axons with smaller diameters are particularly susceptible to conduction block and the irritative, ephaptic, consequences of demyelination and their involvement in the demyelination process caused by MS in the brainstem might underlie the various clinical manifestations of SPS. The adoption of SPS in clinical practice could be useful to improve symptomatic treatments in a significant proportion of patients with MS, possibly limiting the adverse events produced by polypharmacotherapy.

## Narrow Concept of Spasticity

Clinical neurology generally refers to spasticity as a well-defined clinical sign characterized by a velocity-dependent increase of muscle tone (hypertonus) that can be objectivated during the neurological examination (1, 2). This hypertone is generally believed to follow the loss of inhibitory control that the corticospinal tract exerts over spinal tonic stretch reflexes, derived from its degeneration or interruption at different levels due to demyelinating lesions (3). The clinical practice of multiple sclerosis (MS) teaches us that spasticity is not only a sign to be objectified, but it can be better captured taking into account also the subjective complaints or sensations of altered muscle tone and pain occurring during daily activities. It is usually reported by patients and its severity can be described by subjective and objective items reported, respectively, in the Numeric Rating Scale (NRS, variable on a scale of 0–10, where 0 is no spasticity and 10 is the worst possible spasticity), and in the Ashworth Scale (AS, variable from 0—no spasticity to 4—affected parts rigid in flexion or extension) (2), among other scales. According to a classical model trying to provide a plausible explanation for all clinical manifestations typically accompanying spasticity in MS, muscle tone increase caused by corticospinal tract demyelination induces a cascade of downstream effects, giving rise to spasms, cramps, fatigue, tremor, pain, and sleep disturbances. Similarly, the overexcitability or reduced activity of bladder detrusor can cause incontinence or urinary retention (4, 5). This model implies a proportionality between the severity of spasticity and that of the other downstream symptoms, and the idea that the resolution of spasticity, as the primary upstream event, should improve the other signs and symptoms downstream. Unfortunately, this model is not able to cover all the possible eventualities that recur in clinical practice.

## From Classic Model to the Broad SPS Model

**Figure 1:**
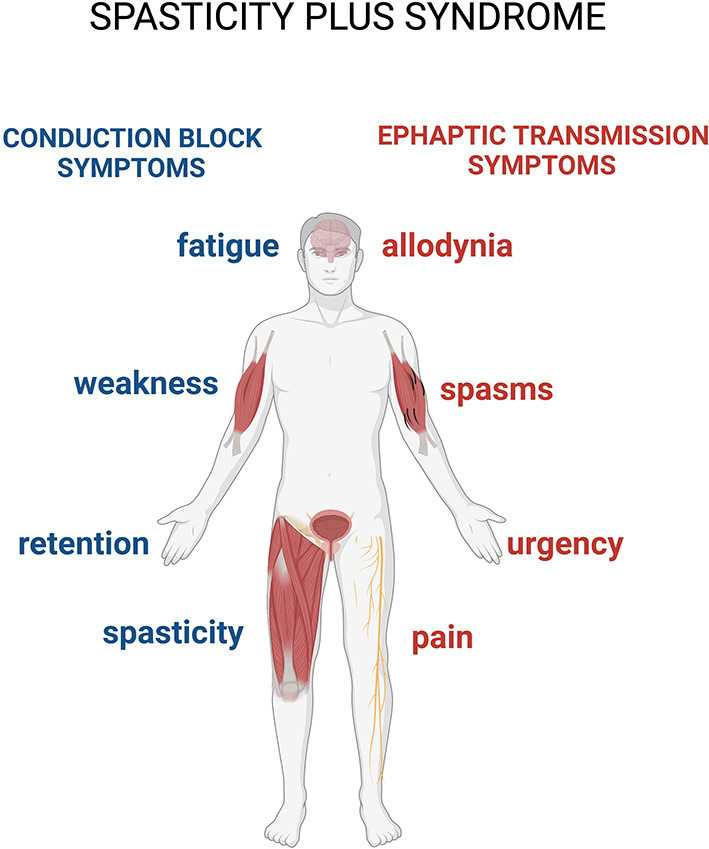
The spasticity-plus syndrome (SPS) model characteristics. The new spasticity-plus syndrome, differently from the classical model, supports the hypothesis that spasticity, triggered by central nervous system (CNS) damage, and other symptoms caused by axon demyelination in terms of conduction block (spasticity, fatigue, weakness, and retention) and ephaptic transmission (spasms, pain, allodynia, and urgency) (see further in the text) are placed on the same level. This model implies that spasticity could be even milder compared to the other associated symptoms.

A syndrome is defined as a variable combination of signs and symptoms forming a distinctive clinical entity and indicative of a particular disease or disorder. An interesting and recent study proposes a new approach to spasticity in MS, introducing the concept of spasticity plus syndrome (SPS), that hypothesizes a new theoretical collocation for this condition (4). According to this proposition, the signs and symptoms accompanying the classically defined spasticity are not connected in a sequential manner after hypertone occurrences in patients with MS, but constitute, along with the so-defined spasticity, a cluster of clinical manifestations, all sharing one specific underlying pathophysiology (4). The novelty of this approach is that it allows putting spasticity and other symptoms (pain, allodynia, bladder dysfunction, fatigue, and sleep disturbances) on the same level (Figure 1).

In the pharmacological management of the various clinical manifestations of MS, many different molecules are generally used as symptomatic agents, alone or in combination. These include anticonvulsants (for example, gabapentin, pregabalin, and lamotrigine), antispasmodics (for example, baclofen), and antidepressants (for example, vortioxetine or citalopram), all aiming at targeting one specific symptom. This approach inevitably implies a loss of the overall vision over the disease processes and at the same time increases the risk of therapy side effects, with a potential worsening of the symptoms complained by the patients. Recognition of SPS as a distinct clinical entity implies that a single drug interfering with its underlying pathophysiology may be able to act simultaneously on all the symptoms that are part of it. It also implies that a given treatment can differently affect the various manifestations of the syndrome in a variable manner, depending on the patient-specific characteristics and pharmacological responsiveness (4) (Figure 1). This approach may also help to shift the focus of clinicians from spasticity alone to the whole cluster of symptoms, encouraging comprehensive understanding and management of the SPS in patients with MS. In their article, Fernandez and colleagues suggest that cannabinoid drugs, whose receptors are diffused at all levels of the cortical spinal tract, could have pleiotropic effects on the various signs and symptoms composing the SPS (4).

In this article, we welcome the theoretical frame implied in the SPS hypothesis, provide a unifying model of SPS signs and symptoms generation in patients with MS and, based on recent literature, suggest that cannabinoid derivatives can act even more extensively on the corticospinal tract through unprecedentedly considered mechanisms.

## Corticospinal Tract, Demyelination, and Origin of SPS Features

In humans, the corticospinal tract originates from the primary motor area (BA 4, 65% fibers) and to a lesser extent from the supplementary motor area (BA 6, 25% fibers) and associative somatosensorial cortex (BA 5, 5%). It is divided into a crossed corticospinal pathway, composed of larger diameter fibers that decussate in bulbar pyramids and course along the lateral column in the spinal cord, and in a direct corticospinal pathway, which contains a lower quantity of fibers, that course along the anteromedial column in the spinal cord, decussating on anterior horns (6). From a pathophysiological point of view, a lesion of the upper motor neuron along the corticospinal tract results in both negative deficient manifestations (weakness) and positive irritative signs (spasticity). In patients with MS, neurologists frequently objectify severe spasticity even in the absence of significant weakness. This feature differentiates the clinical presentation of MS from other diseases also damaging the upper motor neuron, such as ischemic stroke. We propose that this phenomenon could be tentatively explained considering the differential sensitivity of large and small axons to the effects of demyelination.

**Figure 2:**
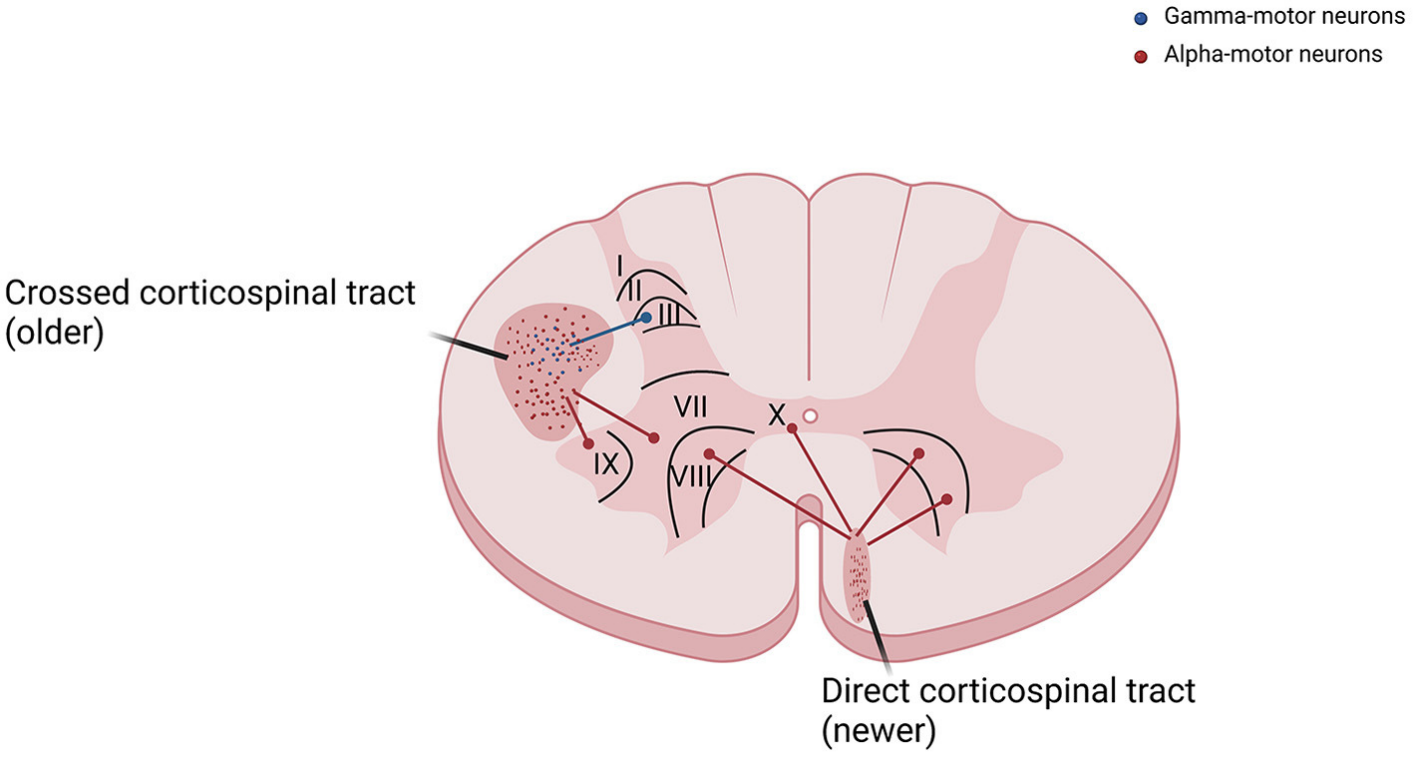
The corticospinal tract is divided into crossed corticospinal tract and direct corticospinal tract. Fibers that reach gamma-motor neurons, responsible for the control of the stretch reflex, are predominantly part of the phylogenetically older lateral or cruciate corticospinal tract. Fibers that reach alpha-motor neurons in the laminae IX and X of spinal cord anterior horns, responsible for the control of voluntary movements, are predominantly part of the newer anterior corticospinal tract.

As already known from neuroanatomy, the corticospinal tract course along with the anteromedial and lateral columns in the spinal cord (3). Axons reach Ia sensory fibers, spinal interneurons, and gamma-motor neurons, and are therefore responsible for the control of the stretch reflex among other phylogenetically preserved spinal reflexes, are predominantly part of the phylogenetically older lateral or cruciate corticospinal tract. On the other hand, fibers that monosynaptically excite alpha-motor neurons in the laminae IX and X of spinal cord anterior horns are predominantly responsible for the control of voluntary movements and are part of the phylogenetically newer anterior corticospinal tract (Figure 2). A similar difference is observed in sensitivity pathways, where axons that conduce pain and visceral sensations have smaller diameters and present a different localization from those that convey tactile and proprioceptive sensitivity (7).

From these considerations, it follows that a selective interruption of the corticospinal lateral tract can give rise to a clinical phenomenon where spasticity would appear even in the absence of weakness. In MS, demyelination of the motor pathway likely involves without distinction both the lateral and the anterior corticospinal tract, thus letting unexplained why spasticity often prevails over weakness in these patients. Our hypothesis is that fibers from the lateral corticospinal tract have a greater sensitivity to the demyelination process than the anterior tract, due to their distinct sizes.

**Figure 3:**
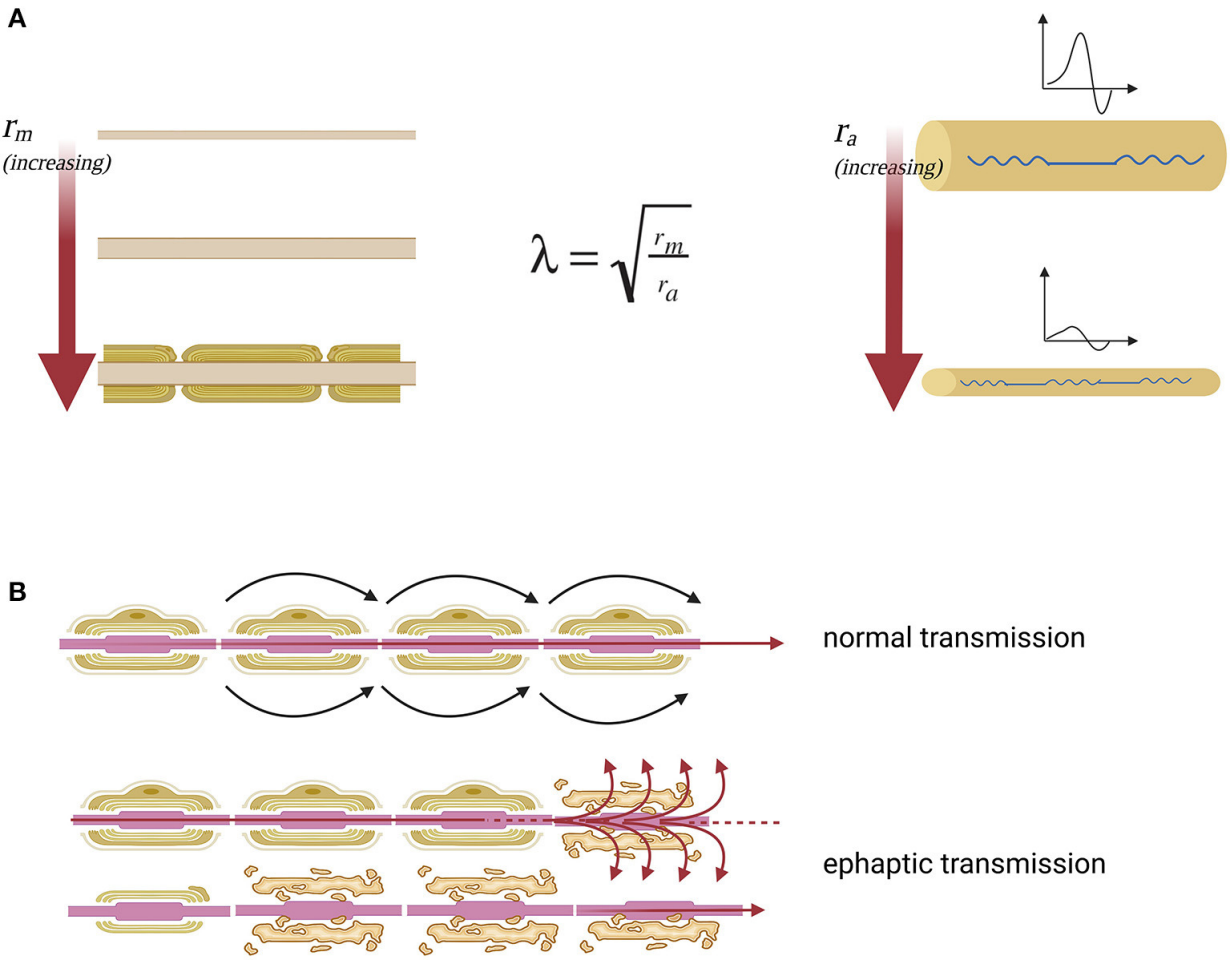
Nerve impulse propagation in axons with normal and reduced diameter. **(A)** Lambda space constant (λ) is an index of how the difference in membrane potential propagates along with the fiber at distance. Moreover, space constant is directly proportional to the membrane resistance (*r*_*m*_), which opposes the escape of ions that must proceed along the axon to transmit the signal and inversely proportional to axial resistance (*r*_*a*_), which opposes the propagation of the action potential along the axon. The membrane resistance increases enormously with the myelination of the fiber, which isolates the axon from current leakage. The axial resistance instead is a direct measure of axonal diameter, so the greater the diameter of the axon, the easier it will be the propagation of excitation at a distance, the smaller the diameter of the axon, the greater the probability that the fiber will suffer a conduction block. From this model, it is clear that the fibers of small diameter are more sensitive to the effects of demyelination because they have high axial resistance. **(B)** The nerve fiber function deficit in the case of SPS can be explained as an effect of ephaptic transmission. In this way when an action potential arises, perturbing the extracellular medium, the excitation is transferred to neighboring axons. Specifically, there is a transverse propagation of excitation from one axon to another, independently of the synaptic junction.

The hypothesized greater sensitivity to demyelination effects of the smaller sized corticospinal later tract could be explained by basic electrophysiology: axons with smaller diameters are more sensitive to conduction block and develop more easily ephaptic transmission to surrounding nerve fibers in response to demyelination. This can be well highlighted by the space constant (λ) of a fiber with electrotonic features, which describes how easily a given perturbation of the resting membrane potential can propagate at a distance (Figure 3A). It can be hypothesized that conduction blocks could be at the basis of the negative, deficitary, and persisting phenomena of SPS such as spasticity, fatigue, weakness, and urinary retention (due to reactivity of the detrusor muscle). Moreover, many other positive, irritative, and recurrent symptoms, such as spasms, pain, allodynia, and urgency, can be better explained in terms of hyperexcitability of demyelinated fibers and secondary ephaptic propagation of excitation. This phenomenon is observed when the depolarizing current of an action potential is forced to pass through a damaged point of an otherwise myelinated tract, thus causing current shunt in the extracellular space and cross-stimulation of other nearby damaged fibers. This type of conduction, which is opposed to synaptic transmission, refers to the fact that a demyelinated axon shares with other demyelinated axons of the same district the same extracellular medium (8) (Figure 3B).

## Axon Demyelination and Voltage-Gated Channelopathy in MS

In physiological conditions, the myelin sheath is essential to ensure high-speed transmission of action potentials along the axons. The ion current associated with action potentials is mediated predominantly by sodium ions flowing through voltage-gated sodium channels (NaV) expressed between two myelinated areas. The intermediate tract between two myelinated areas is called Ranvier's node and expresses the highest density of voltage-gated channel proteins along the entire axon. Since the action potential is renewed at each Ranvier node by “jumping” the myelinated areas, this type of conduction is called saltatory conduction (9).

As reported, demyelination mediated by autoreactive B and T cells causes an action potential propagation disorder along the axons in patients with MS (10). In the case of demyelination, in particular, the absence of voltage-gated channels in demyelinated tracts between Ranvier's nodes causes a progressive amplitude decline of the action potentials during its propagation, possibly resulting in conduction block when the residual amplitude is below the threshold of action potential generation at the next Ranvier's node. In this condition, neurons activate different compensation mechanisms aimed at trying to allow a correct propagation of the action potential at constant amplitude, including ectopic expression of voltage-dependent Na^+^ in demyelinated membrane tracts between Ranvier's nodes. In MS, there is a diffuse expression of both the Nav1.2 and Nav1.6 channels all over the axons (11). Regarding NaV1.6 channels, it is well known that their activation results in persistent Na^+^ influx, imbalance of the Na^+^/Ca^2+^ pump and, intra-axonal Ca^++^ accumulation. Unfortunately, in the long run, these compensatory mechanisms can cause axonal damage by altering the distribution of Na^+^, K^+^, and Ca^2+^ ions across the plasma membrane leading to activation of damaging injury cascades (11, 12) (Table 1). It follows that reducing NaV1.6-mediated hyperexcitability is vitally important to alleviate secondary symptoms in MS (16). Moreover, their wide distribution throughout the CNS makes them crucial targets for the control of SPS symptoms, due to the origin of spasticity from different areas of the brain and spinal cord. A possible class of compounds effective in controlling SPS symptoms could be cannabinoids.

**Table 1:** Multiple sodium channel isoforms are expressed in different tissues and initiate action potentials in neurons, skeletal muscles, and cardiac muscles.

	**Nav1.6**	**NaV1.2**
Physiology	Represent the most important channel across the Ranvier nodes of an adult mature axon (9).	NaV1.2 is normally restricted to immature nodes and unmyelinated fibers (9).
MS/EAE	Preclinical studies observed a reduced expression of Nav1.6 in EAE (9). Contributes to the persistent Na^+^ influx, imbalance of the Na^+^/Ca^2+^ pump and, intra-axonal Ca^++^ accumulation in injured axons in MS. This leads to impaired sodium-calcium exchanger function, may contribute to axonal ephaptic abnormalities, and leads to an activation of damaging injury cascades with consequent neurodegeneration, in the long term (11).	Demyelinated axons are able to express this particular membrane channel (9, 14). The maladaptive role of Nav1.2 expression has been recently investigated in a preclinical study. It has been demonstrated that EAE mice overexpressing Nav1.2, due to a genetic mutation, have exacerbated inflammation-induced neurodegeneration (15).

*Five of the ten known α-subunit isoforms, Nav1.1, Nav1.2, Nav1.3, Nav1.6, and Nav1.8, are found at the CNS nodes in the normal physiological and pathological state (9, 13). Here, we report some features of the most important channels overexpressed on demyelinated axons*.

*Nav, sodium voltage-dependent channel; EAE, experimental autoimmune encephalomyelitis; MS, multiple sclerosis; CNS, central nervous system*.

## Voltage-Dependent Sodium Channels and Cannabinoid

In humans, the endocannabinoid system has a pleiotropic role in the CNS and acts on several elements of the synaptic transmissions. Endocannabinoids interact with different receptors placed at both presynaptic and postsynaptic levels. The main receptors are named CB1 and CB2, among others (17). CB1 is mainly localized at the presynaptic terminal and is involved in synaptic modulation by inhibiting the release of other neurotransmitters (17). The distribution of CB2 receptors in the CNS is concentrated mainly along the sensory pathway, making this receptor a target for neuropathic pain therapy (18). Moreover, CB2 is also localized on microglia and plays a role in immune modulation and neuronal-microglial interaction (17, 19). Converging pieces of evidence are now suggesting that cannabinoids may interact with axonal channels reducing their excitotoxic potential in several pathological conditions. To understand the relationship between the Na^+^/Ca^2+^ pump and cannabinoids, a whole-cell patch-clamp experimental study was conducted on a model of myocardial ischemic damage on rats (20). The authors demonstrated that exposing myocardiocytes to a simulated ischemic medium causes a Na^+^/Ca^2+^ exchanger reversal of function from its physiological role, with an increase of intracellular free Ca^2+^ concentration and a reduction of intracellular free Na^+^ (20). Exposition of myocardiocytes to N-arachidonylethanolamide (anandamide), one of the endocannabinoids, suppresses free Ca^2+^ concentration overload through suppression of the inverted Na^+^/Ca^2+^ exchanger, further demonstrating that this action is specifically mediated by CB2 receptor (20). Moreover, these authors discovered that this effect of anandamide is disease specific, observing that anandamide did not effect on current and intracellular free Ca^2+^ concentration in normal conditions (20). In a second whole-cell patch-clamp study it was studied the relationship between the NaV channels and the cannabinoids in a transgenetic mouse carrying a NaV1.6 gene mutation (16). The authors examined the electrophysiological effects of anandamide and cannabidiol (CBD) in striatal neuron cultures of mutant NaV1.6 compared to wild-type mice. Cannabinoids selectively reduced currents due to pathological expressed NaV1.6 channels on the neuron, influencing both peak current-density and peak resurgent current, thus reducing overall action potential firing of striatal neurons. In particular, cannabinoids did not interfere with the genesis of the action potentials, avoiding a conduction block. Moreover, cannabinoids block the terminal part of repetitive neuronal firing preventing the dispersion of charges through the cell membrane, responsible for ephaptic transmission (16).

In another preclinical study, Ghovanloo and colleagues characterized the electrophysiological effects of CBD on culture cells engineered to expose human NaV (hNaV) channels (21). CBD inhibited hNaV1.1–1.7 currents of HEK293 cells when perfusion reaches therapeutic concentration relevant for humans, thus showing the direct interaction of CBD with NaV channels. They discovered that CBD preferentially stabilizes the inactivated NaV channel states also slowing their recovery from inactivation. Moreover, they also tested the effects of tetrahydrocannabinol (THC) on hNav1.2 channels suggesting that it may represent a different mechanism of inhibition of NaV (21). Recently, an in-depth multidisciplinary study confirmed the functional inhibitory effect of CBD highlighting, by the means of crystallography technique, direct interaction on NaV at the hydrophobic cavity of the channel (22). Finally, another recent electrophysiological study, where the authors demonstrated the ability of CBD to inhibit repetitive action potential firing binding to slow inactivated states of Nav1.8 channels in primary nociceptive neurons from mouse dorsal root ganglia, suggesting that CBD can exert analgesic effects directly inhibiting repetitive firing of primary nociceptors (23).

The finding of a direct effect of cannabinoids on NaV channels further supports their possible use to treat SPS symptoms. The potential efficacy of cannabinoids is also supported by both the widespread diffusion of NaV in the CNS and by their ability to reduce the hyperexcitability of NaV channels, also counteracting ephaptic transmission (20).

## Cannabinoid and SPS

In the previous work, we showed how cannabinoids may have a direct role in axonal pathological conduction in MS and may counteract the symptoms of SPS. This new interpretation integrates what was already stated on SPS (4). In their paper, Fernandez and colleagues mainly focused on the effects of cannabinoids within the CNS, recognizing some points of crucial importance, due to their high concentration of CB1 and CB2 receptors (4). They suggested the brainstem as a critical brain area for the action of cannabinoid-derived drugs, where most of the motor and premotor cortex efferents and sensory afferents converge into relay nuclei and centers responsible for muscle tone and autonomic nervous system functions (4). Here we propose a new insight on the functioning mechanism of the cannabinoid by direct and indirect interaction with demyelinated axons. The importance of cannabinoids is progressively finding a counterpart in clinical practice. A new body of studies showed that THC and CBD are effective in relieving symptoms in patients with MS with moderate to severe spasticity and pain (24). In accord with the SPS model, cannabinoids demonstrate a positive trend in the improvement of bladder function, tremor, fatigue, and sleep disorders in several clinical studies (18, 24). Moreover, recent pieces of evidence suggest that the treatment with THC is associated with a clinically relevant improvement in the Patient Global Impression of Change Scale to quantify and track patient progress and treatment response over time (24). Despite the proven clinical efficacy of synthetic cannabinoids in the treatment of spasticity and pain, the effects of cannabinoids on the other secondary symptoms of MS have not yet been clearly demonstrated and further studies are needed. Moreover, several studies draw attention to the possible effects of synthetic cannabinoid drugs on cognitive functions. In this regard, studies conducted on small cohorts of patients have presented conflicting results. In some cases, showing a cognitive dysfunction associated with cortical thickness reduction in several cortical areas, in other cases showing no significant effect on the cognitive functions of patients with MS (25). In this perspective, a large-scale clinical trial would be needed considering not only spasticity but also all the other secondary symptoms that make up SPS with a particular focus on the potential harm on cognition from chronic use of synthetic cannabinoids in patients with MS.

## Conclusion

Different from the classical approach to spasticity, defined as a neurological sign objectivated by neurologists and influencing other clinical manifestations of MS, there are growing pieces of evidence that lead to a new integrated view of MS symptoms. According to the SPS, the symptoms that commonly accompany the patient with MS (fatigue, insomnia, weakness, spasticity, and urinary disturbances) would constitute a single complex syndrome where all symptoms are placed at the same level. In this context, the use of cannabinoid drugs, thanks to their pleiotropic effects both at the axonal and synaptic level, could play a key role in controlling all clinical manifestations composing the SPS. Cannabinoids usefulness lies both in their proven efficacy in improving all symptoms of SPS and in their safety profile. Furthermore, using a single class of drugs could help to simplify the treatment of these patients who require complex management with polypharmacy by avoiding interactions between multiple drugs. In this regard, a large-scale randomized controlled clinical trial could provide an effective contribution to the involvement of cannabinoids in SPS.

## Data Availability Statement

The original contributions presented in the study are included in the article/supplementary material, further inquiries can be directed to the corresponding author.

## Author Contributions

AB, ED, and DC wrote the manuscript. All authors reviewed the manuscript before submission. All authors contributed to the article and approved the submitted version.

## Funding

This study received funding from Almirall. The funder was not involved in the study design, collection, analysis, interpretation of data, the writing of this article, or the decision to submit it for publication.

## Conflict of Interest

DC is an Advisory Board member of Almirall, Bayer Schering, Biogen, GW Pharmaceuticals, Merck Serono, Novartis, Roche, Sanofi-Genzyme, and Teva and received honoraria for speaking or consultation fees from Almirall, Bayer Schering, Biogen, GW Pharmaceuticals, Merck Serono, Novartis, Roche, Sanofi-Genzyme, and Teva. He is also the principal investigator in clinical trials for Bayer Schering, Biogen, Merck Serono, Mitsubishi, Novartis, Roche, Sanofi-Genzyme, and Teva. His preclinical and clinical research was supported by grants from Bayer Schering, Biogen Idec, Celgene, Merck Serono, Novartis, Roche, Sanofi-Genzyme, and Teva. The remaining authors declare that the research was conducted in the absence of any commercial or financial relationships that could be construed as a potential conflict of interest.

## Publisher's Note

All claims expressed in this article are solely those of the authors and do not necessarily represent those of their affiliated organizations, or those of the publisher, the editors and the reviewers. Any product that may be evaluated in this article, or claim that may be made by its manufacturer, is not guaranteed or endorsed by the publisher.
